# Selective enhancing effect of metal ions on mutagenicity

**DOI:** 10.1186/s41021-016-0049-5

**Published:** 2016-11-01

**Authors:** Nobuyoshi Fujii, Shigemitsu Yano, Kenji Takeshita

**Affiliations:** Safety Evaluation Laboratory, UBE Scientific Analysis Laboratory, Inc., 1978-6, Aza-okinoyama Oaza-kogushi, Ube-shi, Yamaguchi-pref. Japan

**Keywords:** Ames test, Reactive oxygen species, 8-oxoguanine, Oxidative DNA damage, 4-nitroquinoline 1-oxide

## Abstract

**Background:**

We investigated the enhancing effect of metal ions on several mutagens and examined their mechanism of action. We performed the Ames tests on six mutagens, i.e., 2-(2-furyl)-3-(5-nitro-2-furyl)acrylamide, 4-nitroquinoline 1-oxide (4NQO), quercetin, 2-aminoanthracene (2-AA), benzo[a]pyrene, and 3-amino-1,4-dimethyl-5*H*-pyrido-[4,3-*b*]indole, in the presence of five metal ions: Ca(II), Mg(II), Mn(II), Cu(II), and Zn(II).

**Results:**

Cu(II) enhanced the mutagenicity of only 4NQO and reduced the mutagenicity of the other mutagens. Zn (II) enhanced the mutagenicity of only 2-AA. To clarify the mechanism underlying the enhancing effects of Cu(II), we examined the production of reactive oxygen species (ROS) and 8-oxoguanine (8-oxoG), a DNA damage marker, in human lung carcinoma A549 cells. Cu(II) induced a remarkable increase in intracellular ROS and 8-oxoG production in the presence of 4NQO.

**Conclusions:**

Our results suggest that the enhancing effect of Cu(II) and Zn(II) on the mutagenicity of specific mutagens is caused by an increase in ROS.

## Background

Metal ions play crucial roles in biological systems within organisms. However, excessive intake of metal ions causes various disorders, including cancer [1, 2]. Thus, it is important to explore the synergistic effect of metal ions on mutagenicity and to evaluate the environmental and biological risks associated with them. Although some metal ions induce DNA damage, few studies have investigated the effect of metal ions on the mutagenicity of mutagens [3–6]. Because the available data were obtained for a restricted number of mutagens and using different assessment methods, they are not comparable. Therefore, it remains unclear whether metal ions have a selective effect on mutagenicity. This study, aims to investigate the effect of metal ions on mutagenicity. To clarify this, it is necessary that comparative data are obtained using sufficient kinds of metal compounds with the same counter ion and mutagens under identical experimental conditions.

The Ames test (i.e., *Salmonella* mutagenic assay) is a biological assay used worldwide as a preliminary screening method to assess the mutagenic potential of new chemicals and drugs [7, 8]. We performed the Ames test using five metal ions: Ca(II), Mg(II), Mn(II), Cu(II), and Zn(II) and six mutagens: 2-(2-furyl)-3-(5-nitro-2-furyl)acrylamide (AF-2), 4-nitroquinoline 1-oxide (4NQO), quercetin, 2-aminoanthracene (2-AA), benzo[a]pyrene (B[a]P), and 3-amino-1,4-dimethyl-5*H*-pyrido-[4,3-*b*]indole (Trp-p-1). Furthermore, to investigate the mechanism underlying the enhancing effect of metal ions on mutagenicity, we examined the production of reactive oxygen species (ROS), a known cause of oxidative stress, and 8-oxoguanine (8-oxoG), a DNA damage marker, in human lung carcinoma A549 cells [9–11]. We examined and discussed the selective enhancing effect of Cu(II) on mutagenicity that is caused by ROS production.

## Methods

### Materials

The mutagens and metal chlorides used in this study were purchased from Wako Pure Chemical Industries, Ltd., Osaka, Japan and are listed in Table 1. The mutagens were dissolved in dimethyl sulfoxide (DMSO; Kanto Chemical Co., Inc., Tokyo, Japan). The metal chlorides were dissolved in distilled water to prepare the test solutions of 0.25 M, which corresponded to a final concentration of approximately 7.8 mM in the plates. The solutions were diluted to obtain four dose levels of 0.031, 0.063, 0.125, and 0.25 M. S9 was prepared from the livers of male Sprague Dawley rats (Oriental Yeast Co., Ltd., Tokyo, Japan) that had been administered the inducers phenobarbital and 5,6-benzoflavone. 0.5 ml of the S9 mix consisted of 0.05 ml of S9 and 0.45 ml of a cofactor solution containing 4 μmol MgCl_2_, 16.5 μmol KCl, 50 μmol Na_2_HPO_4_/NaH_2_PO_4_, 2 μmol nicotinamide adenine dinucleotide phosphate, 2 μmol nicotinamide adenine dinucleotide, and 2.5 μmol glucose-6-phosphate. Minimal glucose agar plates were purchased from Kyokuto Pharmaceutical Industrial Co., Ltd., Tokyo, Japan.

**Table 1:** Metal chlorides and mutagens used

	Chemical name	CAS number	Purity (%)
Metal chlorides			
	Calcium chloride dihydrate (CaCl_2_・2H_2_O)	10035-04-8	>99
	Magnesium chloride hexahydrate (MgCl_2_・6H_2_O)	7791-18-6	98
	Manganese chloride tetrahydrate (MnCl_2_・4H_2_O)	13446-34-9	99
	Copper(II) chloride dihydrate (CuCl_2_・2H_2_O)	13933-17-0	99
	Zinc chloride (ZnCl_2_)	7646-85-7	98
Mutagenic materials			
	2-(2-furyl)-3-(5-nitro-2-furyl)acrylamide (AF-2)	3688-53-7	>98
	2-aminoanthracene (2-AA)	613-13-8	95
	1,4-dimethyl-5*H*-pyrido[4,3-*b*]indol-3-amine (Trp-p-1)	62450-06-0	97
	Benzo[a]pyrene (B[a]P)	50-32-8	98
	4- nitroquinoline 1-oxide (4NQO)	56-57-5	98
	Quercetin dihydrate (quercetin)	6151-25-3	Practical Grade

### Mutagenicity test

To evaluate mutagenicity, we conducted the Ames test using the pre-incubation method and *Salmonella typhimurium* TA100 (Japan Bioassay Research Center, Tokyo, Japan) as the bacterial strain. We mixed 0.05 ml of the vehicle (DMSO) or the mutagen solutions with 0.1 ml of the metal chloride solutions. Each mixture was assayed in triplicate with and without S9 mix. The solutions were dispensed into sets of test tubes, followed by the addition of either 0.5 ml of S9 mix or 0.1 M phosphate buffer (pH 7.4) and 0.1 ml of bacterial culture. The test tubes were pre-incubated at 37 °C for 20 min, mixed with 2 ml of molten top agar with trace amounts of histidine and biotin, and evenly distributed on the surface of minimal glucose agar plates. After 48 h of incubation at 37 °C, the plates were assessed by counting the number of revertant colonies using a Colony Analyzer CA-11S (System Science Co., Ltd., Tokyo, Japan). Cytotoxicity was assessed by a decrease in bacterial background lawn density.

### Cell culture

A549 cells were seeded at a density of 50000 cells per well in four-chamber slides (Matsunami Glass Industries, Ltd., Osaka, Japan) containing Dulbecco’s modified Eagle’s medium (Gibco Laboratories, Grand Island, NY, USA) with 10 % (v/v) fetal bovine serum. The cells were cultured for 3 days at 37 °C in 5 % CO_2_.

### Production of intracellular reactive oxygen species in A549 cells

A549 cells were incubated with 1 μM 4NQO or AF-2 for 30 min in the absence or presence of 1.25 mM CuCl_2_ in the culture medium, and intracellular ROS were detected using a fluorescent probe and an ROS/RNS Detection Kit (Enzo Life Sciences, Inc., Farmingdale, NY, USA) according to the manufacturer’s instructions. Each well was scanned at a magnification of 200× using a fluorescence microscope. The digitized fluorescence images were captured using an Olympus BX50 System Microscope (Olympus Corporation, Tokyo, Japan) fitted with a QICAM CCD Camera (Nippon Roper K.K., Tokyo, Japan) and sent to a computer. The number of cells and fluorescent optical density of each well, which were measured from an average of at least 200 cells, were analyzed using Image-Pro Plus software (Media Cybernetics, Inc., Rockville, MD, USA).

### Production of intracellular 8-oxoG in A549 cells treated with 4-nitroquinoline 1-oxide

A549 cells were incubated with 1 μM 4NQO for 24 h in the absence or presence of CuCl_2_ (1.25 mM) and labeled with a fluorescein isothiocyanate protein conjugate using an OxyDNA Test (Argutus Medical Ltd., Dublin, Republic of Ireland) according to the manufacturer’s instruction. The fluorescence image of each well was analyzed as described above.

### Statistical analysis

Our data were expressed as the mean number of colonies ± standard deviation for each group. We assessed the differences in colony numbers between the groups using Welch’s *t*-test, and a P value of <0.05 was considered statistically significant.

## Results

### Mutagenicity of metal ions without mutagens

To investigate the effects of metal ions on mutagenicity, we performed the Ames tests for five metal ions, as shown in Table 2. The number of revertant colonies did not increase by the addition of metal ions. Cytotoxicity was not observed at any dose under the conditions tested. Therefore, Zn(II), Mg(II), and Mn(II) can be precipitated as their insoluble phosphates in orthophosphate; however, no precipitation was observed under these conditions.

**Table 2:** Revertant colonies of *Salmonella typhimurium* TA100 with metal ions

With or without S9 mix	Concentration of tested metal solution (M)	Number of His + revertants/plate				
		Ca(II)	Mg(II)	Mn(II)	Cu(II)	Zn(II)
−S9 mix	0	94 ± 10				
	0.031	100 ± 28	93 ± 13	90 ± 9	84 ± 8	89 ± 14
	0.063	79 ± 11	96 ± 9	92 ± 8	80 ± 11	95 ± 21
	0.125	76 ± 5	110 ± 11	100 ± 3	80 ± 17	89 ± 7
	0.25	72 ± 4	87 ± 7	69 ± 6	91 ± 12	99 ± 13
+S9 mix	0	109 ± 8				
	0.031	107 ± 10	112 ± 17	125 ± 21	76 ± 13	104 ± 12
	0.063	124 ± 11	110 ± 12	121 ± 9	70 ± 7	95 ± 10
	0.125	114 ± 9	110 ± 8	103 ± 19	66 ± 3	109 ± 11
	0.25	111 ± 22	115 ± 20	114 ± 8	87 ± 4	117 ± 22

### Effect of metal ions on mutagenic activity of mutagens

To investigate the effect of metal ions on the mutagenicity of the mutagens, we performed the Ames test with the mutagens in the presence of metal ions, as shown in Table 3. The presence of Ca(II), Mg(II), or Mn(II) did not increase the number of revertant colonies, whereas the presence of Cu(II) and Zn(II) induced an increase in the number of colonies.

**Table 3:** Revertant colonies of *Salmonella typhimurium* TA100 with metal ions and mutagens

With or without S9 mix	Mutagenic compounds (μg/plate)	Concentration of tested metal solution (M)	Number of His^+^ revertants/plate				
			Ca(II)	Mg(II)	Mn(II)	Cu(II)	Zn(II)
−S9 mix	-	0	92 ± 15				
	4NQO	0	266 ± 13				
	(0.02)	0.0039	84^a^ ± 11	277 ± 27	252 ± 15	373 ± 73	233 ± 30
		0.016	99^a^ ± 11	236 ± 28	256 ± 23	635 ± 50	246 ± 23
		0.063	86^a^ ± 17	261 ± 22	157^a^ ± 10	619 ± 31	247 ± 14
		0.25	60^a^ ± 9	230 ± 23	70^a^ ± 8	549 ± 52	264 ± 24
	AF-2	0	334 ± 31				
	(0.002)	0.0039	168^a^ ± 24	312 ± 12	324 ± 43	289 ± 26	286 ± 2
		0.016	136^a^ ± 27	280 ± 12	332 ± 48	206 ± 7	262 ± 30
		0.063	119^a^ ± 16	316 ± 11	240 ± 22	229 ± 18	276 ± 15
		0.25	89^a^ ± 12	238* ± 27	109^a^ ± 18	274 ± 22	280 ± 16
	Quercetin	0	235 ± 18				
	(50)	0.0039	79^a^ ± 14	226 ± 37	233 ± 7	90^a^ ± 14	166 ± 23
		0.016	84^a^ ± 10	243 ± 19	179^a^ ± 15	68^a^ ± 10	167 ± 14
		0.063	75^a^ ± 3	249 ± 26	124^a^ ± 22	64^a^ ± 3	157 ± 15
		0.25	58^a^ ± 19	223^a^ ± 14	73^a^ ± 3	63^a^ ± 19	124 ± 6
+S9 mix	-	0	121 ± 39				
	2-AA	0	209 ± 31				
	(0.2)	0.0039	258 ± 30	218 ± 13	186 ± 16	259 ± 25	206 ± 12
		0.016	220 ± 6	229 ± 5	200 ± 16	93^a^ ± 7	236 ± 5
		0.063	269 ± 43	226 ± 19	227 ± 14	71^a^ ± 14	239 ± 19
		0.25	286^a^ ± 21	204^a^ ± 1	232 ± 10	62^a^ ± 6	512 ± 25
	B[a]P	0	196 ± 11				
	(0.5)	0.0039	184 ± 15	188 ± 55	161 ± 20	107 ± 13	203 ± 15
		0.016	187 ± 8	169 ± 13	160 ± 9	75^a^ ± 19	159 ± 11
		0.063	196 ± 7	178 ± 18	180 ± 39	54^a^ ± 14	187 ± 25
		0.25	197^a^ ± 32	179 ± 15	158^a^ ± 15	61^a^ ± 33	140^a^ ± 30
	Trp-p-1	0	215 ± 9				
	(0.1)	0.0039	197 ± 14	211 ± 31	149 ± 18	129 ± 6	167 ± 8
		0.016	206 ± 4	219 ± 43	169 ± 4	89^a^ ± 4	176 ± 4
		0.063	191 ± 17	195 ± 25	174 ± 18	78^a^ ± 20	164 ± 15
		0.25	139^a^ ± 2	173^a^ ±15	145 ± 15	73^a^ ± 6	146 ± 18

Values represent the mean ± SD (*n* = 3)

^a^indicates cytotoxicity

Metal solution was used in amounts of 0.1 ml per plate

**Fig. 1:**
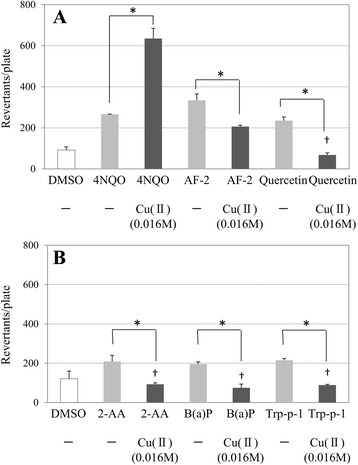
The effect of Cu(II) on mutagenic activity in *Salmonella typhimurium* TA100. Revertant colonies induced by: (**a**) 4NQO (0.02 μg/plate), 2-(2-furyl)-3-(5-nitro-2-furyl)acrylamide (AF-2; 0.002 μg/plate), and quercetin (50 μg/plate) without S9 mix; (**b**) 2-aminoanthracene (2-AA; 0.2 μg/plate), benzo[a]pyrene (B[a]P; 0.5 μg/plate), and 3-amino-1,4-dimethyl-5*H*-pyrido-[4,3-*b*]indole (Trp-p-1; 0.1 μg/plate) with S9 mix. The concentration of Cu(II) solution tested is shown in parentheses. Values represent the mean ± standard deviation (*n* = 3). ^*^Significant increase as determined using Welch’s *t*-test (*P* < 0.05). “†” indicates cytotoxicity

**Fig. 2:**
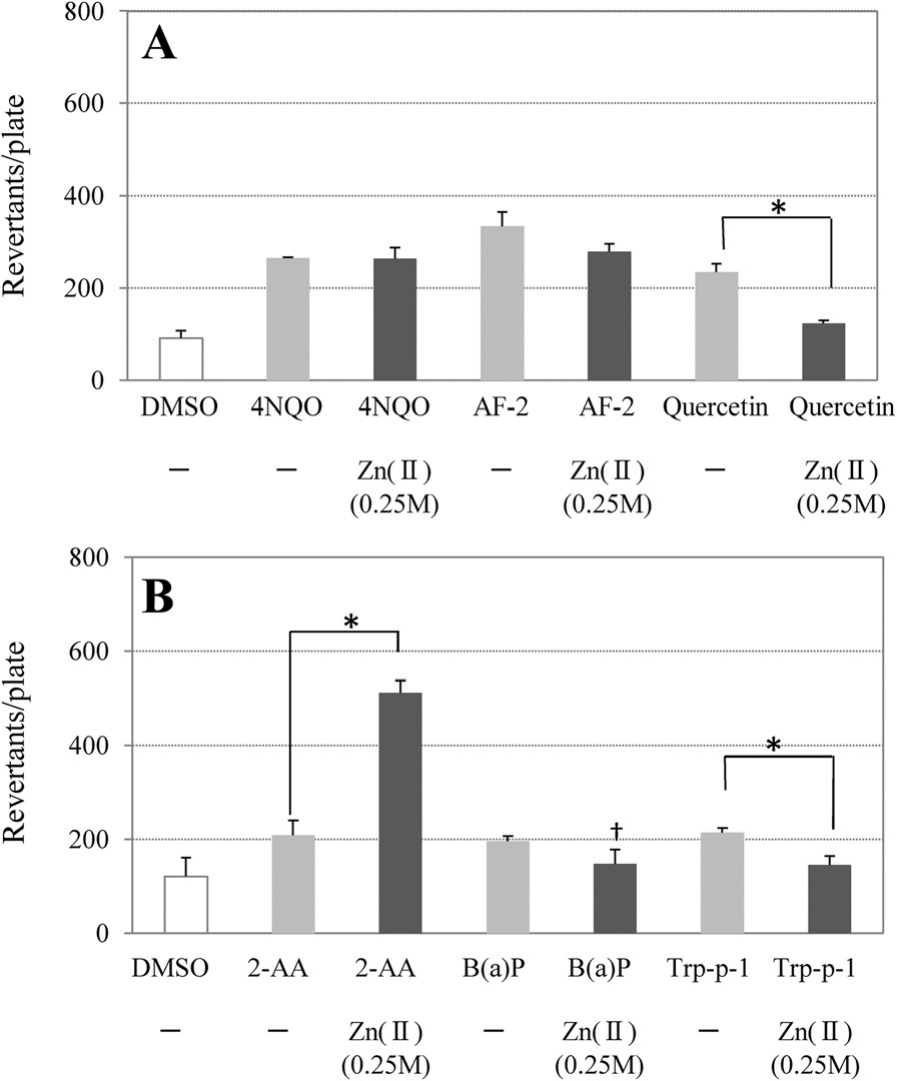
The effect of Zn(II) on mutagenic activity in *Salmonella typhimurium* TA100. Revertant colonies induced by: (**a**) 4-nitroquinoline 1-oxide (4NQO; 0.02 μg/plate), 2-(2-furyl)-3-(5-nitro-2-furyl)acrylamide (AF-2; 0.002 μg/plate), and quercetin (50 μg/plate) without S9 mix; (**b**) 2-aminoanthracene (2-AA; 0.2 μg/plate), benzo[a]pyrene (B[a]P; 0.5 μg/plate), and 3-amino-1,4-dimethyl-5*H*-pyrido-[4,3-*b*]indole (Trp-p-1; 0.1 μg/plate) with S9 mix. The concentration of Zn(II) solution tested is shown in parentheses. Values represent the mean ± standard deviation (*n* = 3). ^*^Significant increase as determined using Welch’s *t*-test (*P* < 0.05). “†” indicates cytotoxicity

The enhancing effect of Cu(II) is represented in Fig. 1. The addition of 0.016 M of Cu(II) induced a two-fold increase in the number of revertant colonies exposed to 4NQO without S9 mix. Cu(II) reduced the number of revertant colonies in the presence of the other mutagens. The enhancing effect of Zn(II) is represented in Fig. 2. More than two-fold increase in the number of revertant colonies was induced by 0.25 M of Zn(II) in the presence of 2-AA with S9 mix. Zn(II) did not induce this increase in the presence of the other mutagens.

As indicated in Table 3, all metal ions induced cytotoxicity in the presence of the mutagens at certain concentrations. Precipitates were not observed at any dose under the conditions tested.

### Effect of Cu(II) on the intracellular production of reactive oxygen species in human A549 cells

**Fig. 3:**
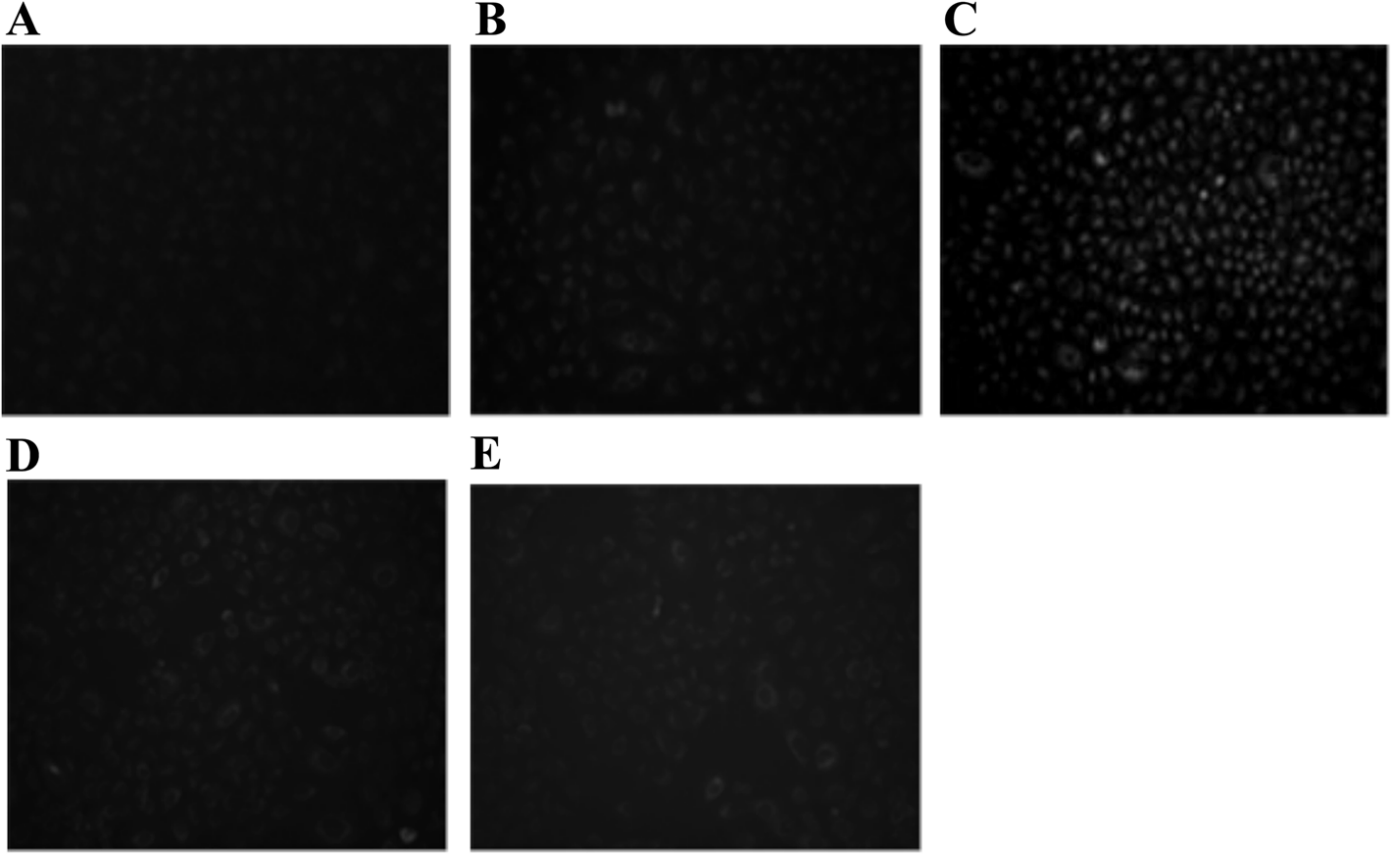
Generation of reactive oxygen species in A549 cells treated with or without 4-nitroquinoline 1-oxide (4NQO) or 2-(2-furyl)-3-(5-nitro-2-furyl)acrylamide (AF-2) in the presence or absence of Cu(II). A549 cells were exposed for 30 min to: (**a**) vehicle; (**b**) 4NQO (1 μM); (**c**) 4NQO (1 μM) and Cu(II) (1.25 mM); (**d**) AF-2 (1 μM); (**e**) AF-2 (1 μM) and Cu(II) (1.25 mM). Intracellular reactive oxygen species were detected using a ROS/RNS Detection Kit (Enzo Life Sciences, Inc., Farmingdale, NY, USA). Each slide was scanned at a magnification of 200× using a fluorescence microscope

**Fig. 4:**
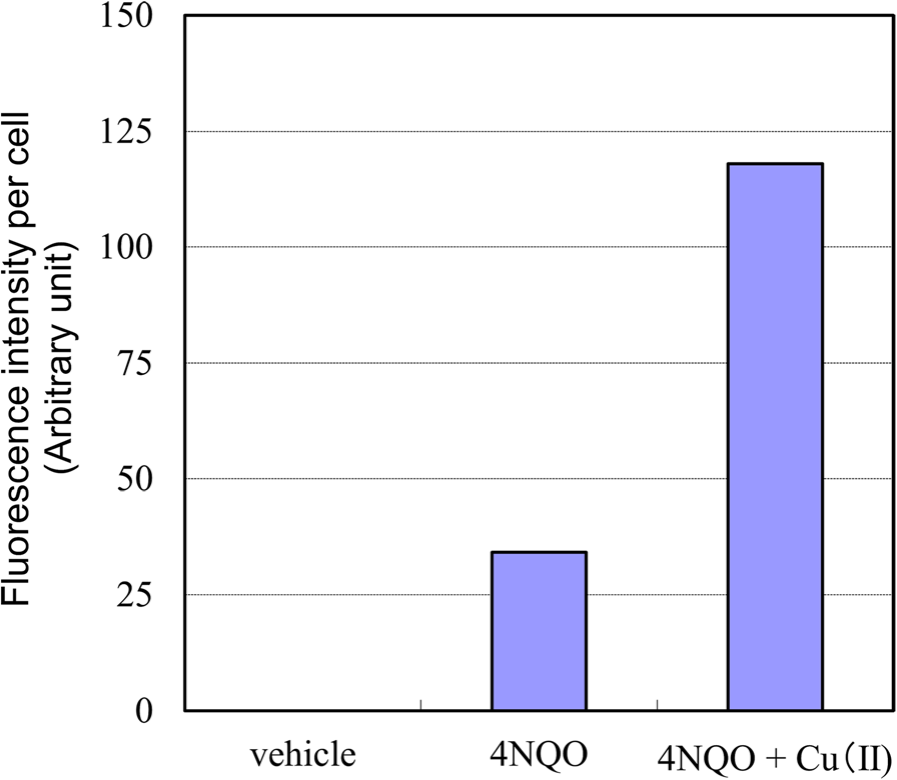
Comparison by digital image analysis of reactive oxygen species production in A549 cells treated with 4-nitroquinoline 1-oxide (4NQO) in the presence or absence of Cu(II). Fluorescence intensities of A549 cells treated with (**a**) vehicle, (**b**) 4NQO (1 μM), (**c**) 4NQO (1 μM) and Cu(II) (1.25 mM) were estimated using Image-Pro Plus software (Media Cybernetics, Inc., Rockville, MD, USA). The results of at least 200 cells were averaged

To investigate the mechanism underlying the enhancement of mutagenicity by Cu(II), we examined intracellular ROS production in A549 cells. Figure 3 demonstrates ROS production in cells exposed to 4NQO and AF-2 in the presence or absence of Cu(II). ROS production in cells exposed to AF-2 was not remarkably promoted by Cu(II), whereas ROS production in cells exposed to 4NQO was increased by Cu(II). The fluorescence image analysis indicated that intracellular ROS production per cell was remarkably increased by Cu(II), as shown in Fig. 4.

**Fig. 5:**
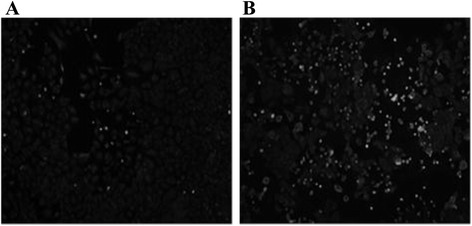
Intracellular production of 8-oxoG in A549 cells treated with 4-nitroquinoline 1-oxide (4NQO). A549 cells were incubated with (**a**) 4NQO (1 μM), (**b**) 4NQO (1 μM) and Cu(II) (1.25 mM), and stained using an OxyDNA Test (Argutus Medical Ltd., Dublin, Ireland). Each slide was scanned at a magnification of 200× using a fluorescence microscope

To confirm this effect of Cu(II), we examined 8-oxoG production, which is a characteristic of oxidatively generated DNA damage, in A549 cells. Figure 5 shows 8-oxoG production by cells exposed to 4NQO in the presence or absence of Cu(II). Clearly, Cu(II) induced a significant increase in 8-oxoG production.

## Discussion

To investigate the effect of metal ions on mutagenicity, we performed systematic experiments. We conducted the Ames test using five metal ions and six mutagens. It is known that 2-AA, B[a]P, and Trp-p-1 require the activation of CYP enzyme, whereas AF-2, 4NQO, and quercetin do not. The mutagenicity of the metal ions examined in this study was negligible at the concentrations tested either with or without S9 mix, as shown in Table 2. This suggests that the metal ions are unable to induce mutagenicity in isolation. The Ames test revealed that Cu(II) has a selective enhancing effect on 4NQO alone, as shown in Table 3 and Fig. 1. Moreover, Zn(II) exhibited an enhancing effect on 2-AA alone, as shown in Fig. 2. Conversely, Ca(II), Mg(II), and Mn(II) did not increase the mutagenicity of the mutagens tested. In Table 3, the number of revertant colonies in several columns was similar to that without metal ions; additionally, cytotoxicity was indicated for those columns. Decreases in the bacterial background lawn density were observed at those columns and the size of revertant colonies was smaller than that without the metal ion. It is presumed that the weak cytotoxicity by metal ions and decrease in the number of revertant colonies were induced at higher doses.

To investigate the mechanism underlying the mutagenicity enhancing effect of Cu(II), we undertook the direct detection of intracellular ROS in A549 cells. As shown in Figs. 3 and 4, Cu(II) exhibited a specific enhancement of ROS production in cells exposed to 4NQO. To confirm this effect on intracellular oxidative stress, we examined 8-oxoG production, a marker of oxidatively generated DNA damage. As shown in Fig. 5, 8-oxoG production in cells exposed to 4NQO was increased by Cu(II). ROS induce the oxidation of guanine to 8-oxoG, which causes the mutation (GC → TA) during DNA repair [12, 13]. This suggests that the enhancing effect of Cu(II) on the mutagenicity of 4NQO is because of an increase in ROS production.

As discussed above, Cu(II) exerts a selective effect on the mutagenicity of 4NQO. Reportedly, metabolites of tryptophan, 2-nitropropane, and catechols increase oxidative DNA damage in the presence of Cu(II) [4, 14, 15]. Although copper is an essential trace element *in vivo* that plays many important roles in enzymatic activity and in the maintenance of chromatin structure [16], copper ions induce DNA cleavage in the presence of a reductant [17–19]. In general, redox-active metal ions, such as iron and copper ions, induce ROS formation through the Fenton reaction in biological systems [20, 21]. Furthermore, copper ions exhibit a high affinity for DNA, in which they preferentially approach consecutive guanines [22–24]. Therefore, the outcome of their promotion of oxidative damage depends on the DNA sequence [25, 26]. Together, these observations using plasmid or extracted DNA suggest that copper ions promote ROS production, thereby inducing DNA damage.

Although it is unclear whether the damage caused by copper ions induces mutations in organisms, the effect of Cu(II) on the mutagenicity of another mutagen could be explained by this mechanism. However, this does not explain the selectivity of Cu(II) for 4NQO; this selectivity is independent of CYP enzymes because 4NQO underwent the Ames test without S9 mix. In Fig. 1, Cu(II) appeared to enhance the mutagenic potency of 4NQO. However, in the presence of the other mutagens, Cu(II) appeared to reduce the mutagenic potency of those agents. This observation suggested that the mechanism underlying the effect of Cu(II) for 4NQO is different from that for the other mutagens.

4NQO, a potent chemical carcinogen, is metabolically converted to 4-hydroxyaminoquinoline 1-oxide (4HAQO) by nitroreductase in microbes and mammalian tissues [27, 28]. 4HAQO binds to DNA after undergoing catalysis by seryl-tRNA synthetases, which elicits its carcinogenicity [29].

Reportedly, 4HAQO cleaves isolated DNA in the presence of Cu(II), but not in the presence of Mn(II), Mn(III), Fe(II) or Fe(III). And because bathocuproine, a Cu(I)-specific chelator, inhibits the DNA damage, it is suggested that the DNA damage induced by 4NQO in the presence of Cu(II) is caused by Cu(I)-peroxide complex or some other copper-oxygen complex [30]. This is one possible mechanism by which Cu(II) selectively enhances the mutagenicity of 4NQO in the Ames test.

Another possible mechanism is that Cu(II) activates some process in 4NQO metabolism. Cu(II) may change the activity of an enzyme, that is not contained in S9 mix., such as seryl-tRNA synthetases in the Ames test.

Furthermore, Zn(II) exerted a selective enhancing effect on 2-AA, which occurred at a higher concentration than Cu(II) and in the presence of S9 mix. It can be speculated that Zn(II) potentiates the metabolic activation of 2-AA by an enzymatic system involving CYPs.

As shown in Fig. 1, Cu(II) appeared to reduce mutagenic potency, except for 4NQO. The decrease in the mutagenicity by metal ion has been reported in Ames test [5].

Reportedly, copper, selenium and zinc inhibit DNA adduct formation of AFB1 [31]. One of the causes of the decrease in the mutagenicity may be inhibition of a mutagen activation process by the metal ions.

Another cause of the decrease in the mutagenicity may be the cytotoxicity of metal. Reportedly, mutagenicity is reduced by the cytotoxicity of metal in Ames test [32]. Furthermore, Cu(II) enhances the cytotoxicity of mutagen which alone exerts no apparent cytotoxicity [33]. The decrease in the number of revertant colonies may be induced by the cytotoxicity of Cu(II) in Fig. 1.

## Conclusion

Our study provides evidence that certain metal ions have the ability to enhance or to reduce the mutagenicity of specific mutagens. This mutagenicity varied with metal ions, and particular metal ions demonstrated different selective effects for different mutagens. This suggests that the risk posed by the mutagens at non-mutagenic doses can be increased by the presence of specific metal ions.

## Abbreviations

2-AA, 2-aminoanthracene; 4HAQO, 4-hydroxyaminoquinoline 1-oxide; 4NQO, 4-nitroquinoline 1-oxide; 8-oxoG, 8-oxoguanine; AF-2, 2-(2-furyl)-3-(5-nitro-2-furyl)acrylamide; AFB1, aflatoxin B1; B[a]P, benzo[a]pyrene; CYP, Cytochrome P450; DMSO, dimethyl sulfoxide; ROS, reactive oxygen species; Trp-p-1, 3-amino-1,4-dimethyl-5*H*-pyrido-[4,3-*b*]indole
